# Editorial

**Published:** 1913-02

**Authors:** 


### EDITORIAL

The Business Office of the *Journal-Record* is 23 West Alabama Street.

The Editorial Office is 1014-15 Atlanta National Bank Building.

Address all Business Communications to *Journal-Record of Medicine*, 23 West Alabama Street.

Make remittances payable to *The Journal-Record of Medicine*.

On matters pertaining to the Editorial and Original Communications, address Edgar G. Ballenger, M. D., Atlanta, Ga.

Reprints of Original Articles will be furnished at cost price. Requests foı the same should always be made in THE MANUSCRIPT.

We will present, postpaid, on request to each contributor of an original article, twenty (20) marked copies of *The Journal-Record of Medicine* containing such article.

### MINISTERS SHOULD DEMAND PREMARITAL CERTIFICATES OF HEALTH

The danger of gonorrhoea and syphilis being carried by man to the woman he is about to marry is usually the result of ignorance or carelessness. There is every reason why restraint should be exercised in granting a marriage license or in performing the ceremony. The stand taken recently by a minister in Chicago, who declared he would henceforth demand a certificate of health from a reputable physician before he would perform the marriage ceremony should open the way for a crusade that could do an immense amount of good.

We all realize full well the futility of attempts at passing legal measures to demand a certificate of health before marriage, and yet we know equally well the necessity of some measure to lessen the innocent suffering and disease of unsuspecting brides. The trouble with legal measures is to find sufficiently plastic rules that would determine who is "fit" and who is "unfit." The wide degree of latitude offered physicians and ministers would be the commendable feature of such a plan as the one suggested. Those in good health would no more mind the examination than they would the one demanded by insurance companies. Only the diseased could object and it would probably make them more careful than they are at present. The groom should be required to present a physician's certificate stating that he is free from gonorrhoea and syphilis. Though not relevant to this particular report, we would recommend furthermore that advanced epilepsy, tuberculosis and cancer be regarded in the same manner in order to prevent the spread or transmission of these diseases. At first it might be well not to require a health certificate of the bride for the cogent reason that disease is much less frequently carried by her than the groom.

While the deterrent effect might not be as great as desired, the educational feature would more than make up for any other shortcomings the method might have. Mothers, fathers, brides and grooms would all concern themselves as to "fitness" and good health and would soon realize certain dangers that are not now dreamed of by some.

While a certain percentage would avail themselves of justices of the peace, if he did not demand a certificate of health, the evasion of the medical examination would soon arouse suspicion and make such marriages unpopular. Atlanta could well afford to provide means whereby those unable to pay for an examination could have it done by the regular city physicians. No reasonable person could say that the romance of marriage would be affected by such a certificate demanded for all. It would not cast a reflection upon either party. If all the ministers of the country would demand a premarital certificate of health we would soon have a state of affairs far better than any legal measures could ever provide.

In answer to the criticism of this plan by a minister we quote an extract from an editorial of the Journal of the American Medical Association, April 6th, 1912.

"One preacher in particular is quoted as waxing eloquent in the denunciation of what he was pleased to call "health certificate marriage." Said he: "If you would rob the holy marriage rite of its sanctity, if you would divest a sacred custom of its beauty and holiness, if you would make the union of two souls a commercial transaction, then establish a custom which will permit the physician and his science to stand between two hearts that are drawn to each other." At the risk of spoiling this perfervid utterance, we fain would take the liberty of paraphasing it, thus: If you would rob the holy marriage rite of its present capacity of permitting the infection of your sisters and your daughters with loathsome diseases, if you would divest a sacred custom of its potentiality for perpetuating epilepsy and idiocy, if you would make the union of two souls synonymous with the union of two clean bodies, then establish a custom which will permit science to stand as a faithful guardian of health and happiness over the two hearts that are drawn to each other!"

